# Prevalence of vitamin D deficiency and association with parathyroid hormone

**DOI:** 10.1515/almed-2021-0078

**Published:** 2022-01-05

**Authors:** Alejandro José Ravelo Marrero, Carlos Antonio Guillén Astete, Miriam Menacho Román, Marta Rosillo Coronado, José Manuel Del Rey Sánchez, Ana Gómez Lozano, María Andreína Terán Tinedo, Mónica Vázquez Díaz, Ignacio Arribas Gómez

**Affiliations:** Clinical Chemist, Hospital Ramón y Cajal, Madrid, Spain; Rheumatologist, Hospital Ramón y Cajal, Madrid, Spain

**Keywords:** 25-hydroxyvitamin D, parathyroid hormone, prevalence

## Abstract

**Objectives:**

We evaluated the prevalence of 25-hydroxyvitamin D (25-(OH)D) deficiency in our setting according to season, sex, and age. We also studied the association with parathyroid hormone (PTH) levels.

**Methods:**

The study population comprised all patients with requests for assessment of 25-(OH)D between January 1 and December 31, 2018, as registered in the database of the laboratory information system. Major exclusion criteria were pediatric samples (<18 years) and factors affecting 25-(OH)D and/or PTH levels (i.e., kidney injury, liver disease, PTH disorders).

**Results:**

Among 33,601 patients (24,028 women, 9,573 men), the prevalence of 25-(OH)D deficiency was 48%. Prevalence was greater in males than in females (53% vs. 46%). By age group, deficiency was more prevalent in quartile 1 (Q1, 74–87 years) and less prevalent in quartile 2 (Q2, 60–73 years). By season, deficiency was greater in spring (nonsignificant differences with respect to winter) and lower in summer. The association between 25-(OH)D and PTH was assessed in 9,368 persons. Linear regression analysis showed a weak association (coefficient – 0.303). Multiple logistic regression analysis revealed a significant association between 25-(OH)D deficiency and increased PTH (Odds ratio (OR), 1.63). Other risk factors for increased PTH include female sex (OR, 1.27), season (winter, OR 1.63, spring OR 1.16), and age (quartile 1, OR, 3).

**Conclusions:**

The prevalence of 25-(OH)D deficiency differed according to sex, age, and season of the year. Furthermore, elevation of PTH is mainly influenced by low 25-(OH)D, female sex, season, and age.

## Introduction

In recent years, vitamin D deficiency has given considerable cause for concern among physicians. This is reflected in the marked increase in requests for diagnostic tests and prescriptions for supplements [1]. However, no consensus has been reached on the definition of this deficiency, and review articles on the subject, such as that by Cashman et al. [2], continue to be published. The thresholds for deficiency have been established at serum 25-hydroxyvitamin D (25-(OH)D) concentrations that range from <10 ng/mL to <30 ng/mL depending on the country, health care organization, and scientific society [3].

From a population-based perspective, the US Institute of Medicine (IOM) recommends a dietary reference intake for vitamin D. Using data on bone health as a criterion, they suggest that a serum concentration of 20 ng/mL would meet the needs of 97.5% of the population [4]. Similarly, the European Food Safety Authority set the same threshold as the IOM, although they recognize that available data prevent us from saying whether this would be an achievable objective in half or in the majority of the population [5]*.* However, the UK Scientific Assessment Committee on Nutrition set their cut-off point for this deficiency of <10 ng/mL (<25 nmol/L) based on the increased risk of rickets in children and osteomalacia in adults [6].

Based on a clinical practice approach aimed at the individual patient, expert panels from the Endocrine Society, the National Osteoporosis Foundation, the International Osteoporosis Foundation, and the American Geriatric Society recommend serum concentrations of 25-(OH)D >30 ng/mL (>75 nmol/L), especially in elderly persons [7], [8], [9], [10], [11].

Estimation of the prevalence of 25-(OH)D deficiency depends on factors such as sex, age, season, race, and methodology (study design, technique used to quantify 25-(OH)D), thus leading to broad discrepancies between studies [3, 12], [13], [14], [15], [16]. Recently published estimations of prevalence have been based on national surveys and on standardization of the measurement of 25-(OH)D by the Vitamin D Standardization Program (VDSP) run by the US National Institutes of Health. Therefore, for a VDSP threshold of 25-(OH)D <12 ng/mL, the prevalence was 5.9% in the USA [17], 7.4% in Canada [18], and 13% in Europe, which reached 40% when the cut-off value was set at <20 ng/mL [19].

Attempts to set an optimal value for 25-(OH)D have also been made based on its effect on parathyroid hormone (PTH). This has been defined as the minimum 25-(OH)D concentration necessary to avoid secondary hyperparathyroidism and the resulting osteoporosis [20, 21]. However, it has not been possible to establish a consistent value for 25-(OH)D below which hyperparathyroidism develops. Multiple cross-sectional studies have shown a negative correlation between 25-(OH)D levels and PTH and report contradictory results for cut-off points and a prevalence of increased PTH of 10–33% in patients with hypovitaminosis D [22, 23].

The above observation may be the result of multiple factors, such as differences in the standardization of trials to determine PTH and the heterogeneous nature of the study populations in terms of factors such as race, age, and gender.

The National Health and Nutrition Examination Survey (NHANES) 2003–2004 and 2005–2006 showed significant differences between races for the association between 25-(OH)D and PTH above and below the threshold that is commonly used to define vitamin D deficiency (20 ng/mL), with inverse associations in Whites and Mexican Americans, but not in Blacks [24]*.*

Our study proposes to evaluate the prevalence of vitamin D deficiency in our setting according to season, sex, and age and to study the association with PTH levels.

## Materials and methods

### Patients and design

We performed a cross-sectional observational historical study. The study population comprised all requests of outpatient or primary care for whom assessment of 25-(OH)D between January 1 and December 31, 2018, as registered in the database of the laboratory information system (LIS, Openlab 10.0.43, Nexus). We also collected other data of interest, namely, PTH, calcium (serum and urine), phosphate (serum and urine), creatinine (serum and urine), serum magnesium, total alkaline phosphatase, gamma-glutamyltransferase, lactate dehydrogenase, total bilirubin, and glutamate-pyruvate dehydrogenase.

**Figure 1:**
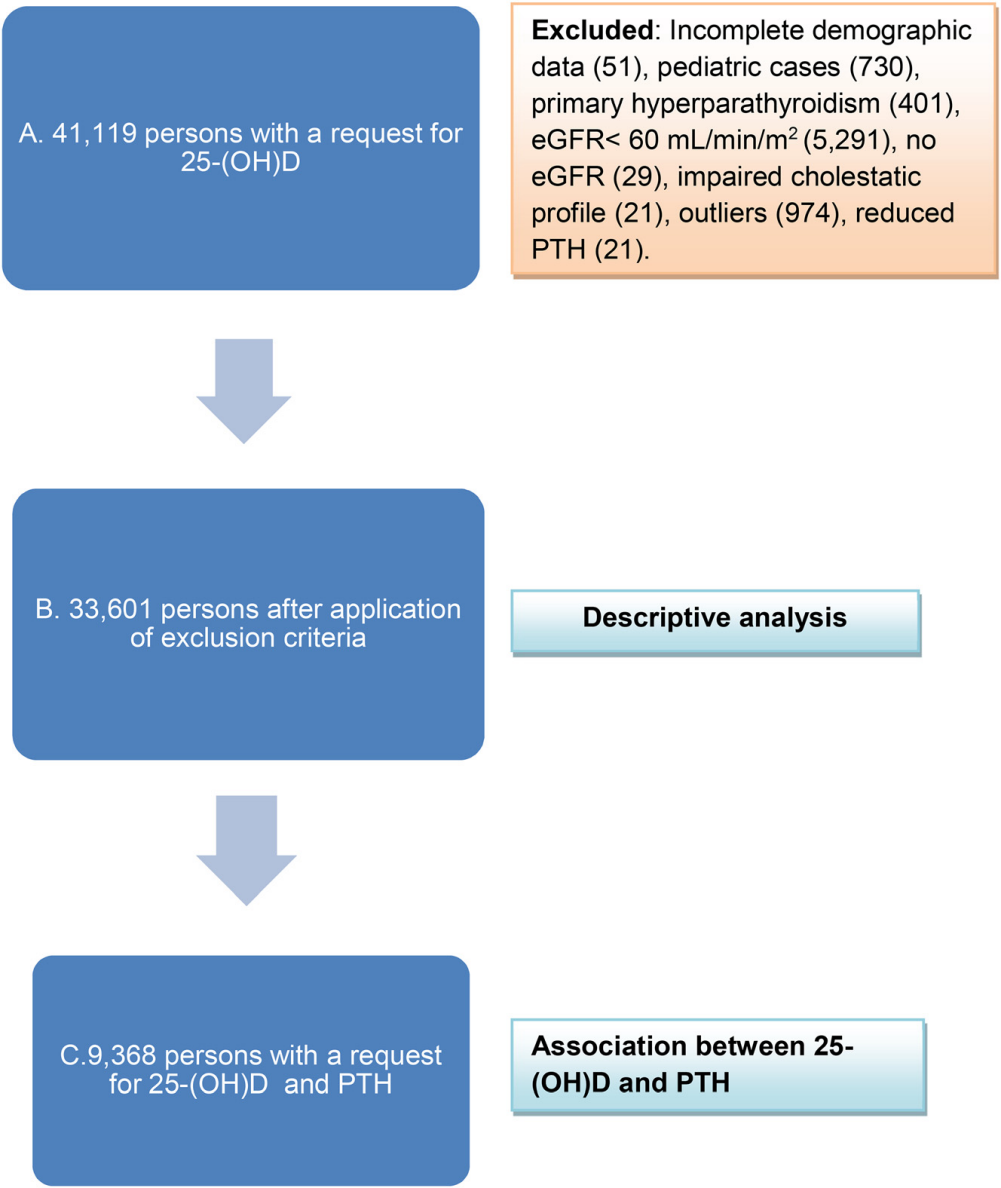
Study participants. The reasons for exclusion and the analyses performed in each group are specified. (A) Persons with a request for 25-(OH) D, (B) after exclusion criteria, and (C) requested for 25-(OH) D and PTH at the same time. 25-(OH) D, 25-hydroxyvitamin D; eGFR, estimated glomerular filtration rate; PTH, parathyroid hormone.

We excluded requests without a date of birth, pediatric samples (<18 years), intensely hemolyzed samples, samples from patients with chronic kidney disease (CKD) ≥ stage 3 (estimated glomerular filtration rate (eGFR) <60 mL/min/1.73 m^2^) [25], patients with suspected primary hyperparathyroidism (PTH >65 pg/mL and calcium >10.3 mg/dL) or hypoparathyroidism (PTH <12 pg/mL) (PTH reference range, 12–65 pg/mL), and patients with liver disease or cholestasis. Similarly, we ruled out atypical values (outliers, i.e., values with a *z*-score less than −3 or greater than 3) for the main parameters, namely, 25-(OH)D, PTH, and calcium (Figure 1A–C). In the case of patients with more than one request, only the first initial determination was included.

### Materials

A series of parameters were measured using the ARCHITECT c16000 analyzer (Abbott) with spectrophotometry by means of colored complexes or enzyme reactions, as follows: serum and urinary calcium, serum and urinary creatinine, serum and urinary phosphate, serum magnesium, total alkaline phosphatase, gamma-glutamyltransferase, total bilirubin, lactate dehydrogenase, and glutamate-pyruvate transaminase. PTH was measured using electrochemiluminescence (intact PTH, STAT [short turnaround time]) in a COBAS e411 analyzer (Roche). 25-(OH)D was measured using microparticle chemiluminescent immunoanalysis in the ARCHITECT i2000sr analyzer (Abbott) and was standardized with respect to the reference method (high-performance liquid chromatography–mass spectrometry [HPLC-MS]) based on the Centers for Disease Control and Prevention Vitamin D Standardization-Certification Program (CDC VDSCP, List Number 5P02). All samples were collected according to the technical specifications of the manufacturer. Cold EDTA tubes were used to preserve stability of PTH.

### Statistical analysis

The descriptive analysis based on available results included a total of 33,601 patients (9,573 men; 24,028 women) (Figure 1). The statistical measures used where the mean ( $\overset{–}{\text{x}}$ ) and standard deviation (SD). The prevalence of vitamin D deficiency was calculated according to sex, age, and season based on the local 25-(OH)D cut-off point (<20 ng/mL).

Differences in the means of the quantitative variables between the two groups were evaluated using the *t* test. Mean differences in 25-(OH)D between the seasons were evaluated using analysis of variance (ANOVA) and a post hoc Scheffé test. This analysis was also carried out for patients whose 25-(OH)D was measured during the last 2 weeks of each season, since variations during this period are more representative of the effect of the season on serum 25-(OH)D levels.

The association between 25-(OH)D and PTH was assessed using regression analysis based on a population of 9,368 patients (Figure 1). The first stage involved a linear regression analysis. The study was completed with a binary multivariate logistic regression analysis between PTH and the predictive variables 25-(OH)D, sex, age, and season. We based our analysis on a complete model including all predictors of interest, as well as 25-(OH)D (age, sex, season), as well as the interaction between these predictors and 25-(OH)D itself. Variables were gradually eliminated one by one, starting with lower significance values (high p value). The model was re-run every time a variable was removed until the final model was obtained, i.e., one containing only statistically significant variables and/or variables with a confounding effect on 25-(OH)D (confounding was defined as the odds ratio (OR) for 25-(OH)D being modified by more than 10% when a variable was removed). Goodness of fit was evaluated using the Hosmer-Lemeshow test. The discriminatory capacity of the model was analyzed using the area under the curve (AUC). Statistical significance was set at p<0.05 (2-sided). The precision of the measurements was based on the 95% confidence interval (95% CI) of the estimations. The statistical analysis was performed using SPSS Version 15.0 (SPSS Inc., Chicago, IL, USA).

## Results

The descriptive statistics for the parameters analyzed in the study population are shown in Table 1. The mean age of the study population was 59 years (58 in men and 60 in women) We observed differences between the means according to sex for PTH, both with values above the reference range for healthy individuals (men, 65.4 pg/mL; women, 70.4 pg/mL) and with respect to 25-(OH)D (men, 20.94 ng/mL; women, 22.75 ng/mL). Sex differences with respect to PTH and 25-(OH)D were statistically significant (p<0.001).

**Table 1:** Distribution of the parameters analyzed in the study population.

	$\bar{x}$	SD	Minimum	Maximum
Age, years	59	18	18	106
25-(OH)D, ng/mL	22	12	1	67
PTH, pg/mL	69	30	12	204
Calcium, mg/dL	9.41	0.39	8.2	10.6
Phosphate, mg/dL	3.32	0.53	1.1	6.7
Magnesium, mg/dL	1.98	0.2	1	3
Calcium/creatinine	0.16	0.09	0	0.8
Tubular phosphate reabsorption, %	82	7.2	17	97

PTH, parathyroid hormone.

Data on the prevalence of 25-(OH)D deficiency according to sex indicated that the condition was more prevalent in men (53% [5,030/9,573])than in women (46% [11,103/24,028]). The overall prevalence was 48% (16,133/33,601).

The prevalence of 25-(OH)D deficiency according to age and season is shown in Table 2. By age quartiles (Q1–Q4), deficiency was more prevalent in Q1 (74–87 years) and less prevalent in Q2 (60–73 years). As for seasons, deficiency was greater in spring and lower in summer. The mean interseasonal difference between spring and summer was 4.07 ng/mL (Table 3). The mean differences in 25-(OH)D between the seasons were significant in the ANOVA, except for the difference between spring and winter. When 25-(OH)D was measured during the last 2 weeks of each season autumn showed the lowest levels of 25-(OH)D. Again, differences in means were significant except for the difference between spring and winter.

**Table 2:** Prevalence of 25-(OH)D deficiency by age quartile and season.

Age quartile		Winter		Autumn		Spring		Summer	
		n	%	n	%	n	%	n	%
Q1	Deficiency	1,257	54	1,026	52	1,427	56	763	51
	(95% CI)		(52–56)		(50–54)		(54–58)		(49–53)
	Total	2,343		1,968		2,569		1,486	
Q2	Deficiency	1,144	46	789	37	1,221	49	487	33
	(95% CI)		(44–48)		(35–39)		(47–51)		(31–35)
	Total	2,467		2,120		2,482		1,497	
Q3	Deficiency	1,248	53	775	43	1,288	54	510	32
	(95% CI)		(51–55)		(41–45)		(52–56)		(30–34)
	Total	2,345		1,786		2,394		1,576	
Q4	Deficiency	1,490	61	861	42	1,385	60	462	26
	(95% CI)		(59–63)		(40–44)		(58–62)		(24–28)
	Total	2,437		2,048		2,311		1,772	
	Deficiency	5,139	54	3,451	44	5,321	55	2,222	35
	(95% CI)		(52–56)		(42–46)		(53–57)		(33–37)
	Total	9,592		7,922		9,756		6,331	

Age quartiles: Q1 (74–87 years), Q2 (60–73 years), Q3 (46–59 years), Q4 (32–45 years); deficiency: 25-(OH)D deficiency.

**Table 3:** Serum concentrations of 25-(OH)D according to season.

Season	Spring	Winter	Autumn	Summer
$\bar{\text{x}}$ , ng/mL	20.95	21.09	22.95	25.04
SD	12.36	12.37	11.98	12.19

The linear regression analysis of vitamin D and PTH revealed a weak association, with a coefficient of −0.303 (95% CI, −0.349 to −0.257; p<0.001).

The association between PTH and the predictive variables 25-(OH)D, sex, age, and season was investigated using multiple logistic regression analysis. In order to rule out interactions between predictive variables, we started with a model that included all possible interactions between independent variables and the main variable (25-(OH)D). The model included variables for which there was no interaction and variables with a double, triple, or quadruple interaction with 25-(OH)D. Nonsignificant interactions were eliminated hierarchically until the final model was achieved (Table 4).

**Table 4:** Logistic regression analysis (vs. PTH).

	Beta coefficient	Adjusted OR	95% CI		p-Value
			Lower	Upper	
25-(OH)D	0.49	1.63	1.37	1.94	<0.001
Female sex	0.24	1.27	1.16	1.39	<0.001
Seasons					<0.001
Spring	0.15	1.16	1.02	1.32	0.02
Winter	0.49	1.63	1.44	1.86	<0.001
Autumn	0.1	1.11	0.96	1.28	0.16
Age quartiles (Q1–Q3)					<0.001
Q1	1.1	3	2.51	3.59	<0.001
Q2	1.08	2.96	2.51	3.49	<0.001
Q3	0.63	1.88	1.59	2.21	<0.001
Age quartile*25-(OH)D					0.002
Q1*25-(OH)D	0.35	1.42	1.09	1.84	0.009
Q2*25-(OH)D	–0.1	0.91	0.71	1.16	0.44
Q3*25-(OH)D	–0.1	0.9	0.71	1.14	0.4
Constant	–1.18	0.31			<0.001

Age quartiles (Q1–Q3): Q1 (74–87 years), Q2 (60–73 years), Q3 (46–59 years), Q4 (32–45 years). OR, odds ratio; PTH, parathyroid hormone.

## Discussion

The prevalence of 25-(OH)D differed according to sex, age, and season of the year. Furthermore, the multivariate analysis showed that age, season and sex affected the association between 25-(OH)D and PTH. We provide results on the prevalence of 25-(OH)D deficiency in a large series of individuals.

The prevalence of vitamin D deficiency in our setting is around 48%, which is similar to that reported in New Zealand [26]. Various published series in Spain report 25-(OH)D deficiency in different age groups (children, postmenopausal women, elderly persons), irrespective of their degree of exposure to sunlight [27], [28], [29], [30], [31], [32].

The estimated prevalence of 25-(OH)D deficiency in Spain is not homogeneous. Studies include very disparate populations (elderly persons, postmenopausal women, children) and wide differences in methods of measuring 25-(OH)D (radioimmunoassay, competitive protein binding, HPLC), which hamper comparison between affected persons.

In their study of the prevalence of hypovitaminosis D in Madrid, Aguado et al. [27] found an 84% prevalence of deficiency in 171 postmenopausal women with rheumatic disease based on a cut-off point of 20 ng/mL. 25-(OH)D was measured using radioimmunoassay. Mata-Granados et al. [29] reported a prevalence of deficiency of 51% (25-(OH)D between 10 and 20 ng/mL) in men and women aged 18–65 years in Cordoba, with determination of 25-(OH)D by HPLC. Quesada et al. [28] reported a 44% prevalence of 25-(OH)D deficiency in untreated postmenopausal women and 29% in treated women. Mean age was 71 years, and the sample was taken from throughout Spain (28–43°N). 25-(OH)D was measured using HPLC. Lips et al. [30] reported a prevalence of 42% in women aged 64 years with osteoporosis (37–43°N) after measuring 25-(OH)D using radioimmunoassay.

In Europe, Cashman et al. [19] recorded serum 25-(OH)D values standardized using the VDSP (measurement by HPLC-MS). Analysis of a total of 55,844 samples of all ages from 14 countries yielded a 40% prevalence of deficiency (cut-off, 20 ng/mL).

In our series, the prevalence of deficiency was greater in males than in females (53% vs. 46%). Consistent with our research, AlQuaiz et al. [33] reported a greater prevalence of deficiency among men in a retrospective study of persons aged 30–75 years (n=2,835 patients) in primary care in Saudi Arabia.

The differences between sexes recorded in our study are consistent with the results of a study on the prevalence of 25-(OH)D deficiency in Chile [34] (latitude 33°S). The authors reported the prevalence of 25-(OH)D deficiency by sex in healthy persons aged 18–89 years (n=1,329) and obtained higher values in males (45.9%). In addition, the differences between males and females were thought to be due to lower exposure to sunlight among men with respect to women, mainly younger women. The main factors underlying this discrepancy are the greater percentage of work outside the home and sedentary lifestyle among males.

Among women menopausia could be a factor to consider when evaluating 25-(OH)D deficiency. According to the scientific literature, the relationship between menopause and hypovitaminosis D has not been shown to have a causal background but rather a mere epidemiological association.

On the one hand, the determination of vitamin D levels is a behavior that is commonly performed in postmenopausal women, at least in a first assessment. Determination in other clinical settings is very rare in developed countries. Then, the association between hypovitaminosis D in postmenopausal women is relatively frequent, first, because it is in this group of patients that this determination is most frequently performed.

The time spent in the sun also decreases with age as people become more sedentary. This factor could also be an indirect factor leading to a link between menopause and hypovitaminosis D. Another known intermediary factor between menopause and vitamin D deficiency is central obesity and malabsorptive syndromes. These conditions also become more prevalent with age and therefore more frequent in the postmenopausal population among males [7, 35, 36].

By season, the prevalence of deficiency in our study was greater in spring (nonsignificant differences with respect to winter) and lower in summer. The presence of interseasonal variation was also confirmed when the comparison was repeated between persons whose 25-(OH)D had been assessed during the last 2 weeks of each season. In this case, deficiency of 25-(OH)D was lower in summer and higher at the end of autumn providing a possible explanation of the low levels of 25-(OH)D during winter months. We hypothesize differences in 25-(OH)D serum concentrations from the end of summer to the end of autumn may be due to higher exposure to sunlight in summer and lack of supplementation during these months.

Our study confirms previously reported seasonal variations in 25-(OH)D throughout the year. Our results agree with those of other studies [37], [38], [39], which show a greater prevalence of 25-(OH)D deficiency in winter and spring. The main discrepancies in our study are found during the season with the lowest prevalence of deficiency (summer vs. autumn in the studies cited above).

Cutaneous synthesis of 25-(OH)D decreases during winter owing to the reduced time of exposure to sunlight compared with summer. Latitude plays a key role, given the considerable differences between regions that lie below and above 37°N, since the latter are less likely to synthesize cutaneous 25-(OH)D during the winter months owing to the incident angle of UV rays [40]. Our region lies at 40°N, and this should be taken into account when evaluating results.

Pereda et al. [37] reported vitamin D deficiency in adult women with rheumatic disease (mean, 53.2 years) in the Spanish region of Almería (latitude 36°N). In a total population of 319 patients (81.5% postmenopausal) not taking 25-(OH)D supplementation, mean vitamin D levels were <30 ng/mL, except in autumn, despite optimal sunlight.

Gozdzik et al. [38] reported seasonal variation in 25-(OH)D deficiency in young adults (18–35 years) in Toronto (latitude 43°N). The authors measured 25-(OH)D over 2 years in autumn and winter and observed mean differences of 6 ng/mL between autumn and winter (21.8–15.8 respectively). The interseasonal differences in their study population depended mainly on intake of vitamin D and skin pigmentation.

In terms of age, the greatest prevalence of 25-(OH)D was found in the oldest patients (Q1: 74–87 years). Consistent with two studies performed in Spain, a high prevalence was recorded in elderly age groups [31, 32]. In both studies, the prevalence was 87% in institutionalized elderly persons [31] and noninstitutionalized persons [32] in Barcelona.

As reported in the literature [20, 21], PTH was used as the main outcome measure owing to the clinical association with 25-(OH)D, despite a weak correlation between both parameters. Our study confirmed this correlation (coefficient, −0.303).

The multivariate analysis revealed a significant association between 25-(OH)D deficiency and increased PTH (OR, 1.63). Other risk factors for increased PTH include female sex (OR, 1.27), season (winter, OR 1.63, spring OR 1.16) and age (Q1, OR, 3).

The effect of female sex on PTH is seen in the observation that plasma PTH levels are higher in women than in men (70.4 pg/mL and 65.4 pg/mL, respectively). These differences in PTH between the sexes are reported elsewhere [41].

The study of interactions between predictive variables (season, sex, age) and PTH ruled out all interactions except for age with 25-(OH)D (p<0.05); therefore, these were excluded from the final model. With respect to the age groups, the only interaction identified with 25-(OH)D was for elderly persons (Q1) (p=0.009). This result suggests a possible effect of age on serum 25-(OH)D levels and should be taken into account when evaluating the association between 25-(OH)D and PTH. Elderly persons have a greater risk of high PTH, and their serum levels of 25-(OH)D are lower than in the rest of the population. When age and 25-(OH)D are taken together only in elderly persons with 25-(OH)D deficiency, PTH increases.

Surprisingly, the effect of age on 25-(OH)D and of 25-(OH)D on PTH is not seen in other older patients, such as those in Q2 (60–73 years). In fact, the lowest prevalence of 25-(OH)D deficiency was recorded in this quartile, probably because they more frequently took supplements than patients in the other quartiles. However, we were unable to demonstrate this.

Concerning season, PTH elevation was mainly associated with winter and spring (autumn was nonsignificant) suggesting seasonality as it occurred in 25-(OH)D. This interseasonal variation of PTH has already been described [42]. Therefore, it should be taken into account when evaluating 25-(OH)D and/or PTH values.

The model was adequately calibrated, as shown by the Hosmer-Lemeshow test (p=0.842). The AUC was 0.656, which is close to the acceptable cut-off point (AUC ≥0.7), thus indicating the low degree of discrimination of the model between patients with normal PTH values and those with values higher than the reference values. This result confirms that 25-(OH)D as a key predictive variable cannot explain a large percentage of variations in PTH (34.4% in our series).

Our study has various strengths. First, the large size of the study population means that our statistical power is considerable. Second, our technique for measuring 25-(OH)D was robust, given that it was standardized by the CDC. This ensured a systematic error of ±5% and an imprecision of <10% with respect to the reference method for determination of the analyte.

The main limitation of our study is its historical design. There may be a bias in the selection of patients, since the prevalence of 25-(OH)D deficiency is calculated based on patients who underwent testing and on the association with PTH in patients who underwent joint assessment of 25-(OH)D and PTH. In an ideal study, both 25-(OH)D and PTH would have been determined in all patients. Furthermore, the design of the study prevented us from distinguishing between patients who took supplements and those who did not.

## References

[1] Crowe FL, Jolly K, MacArthur C, Manaseki-Holland S, Gittoes N, Hewison M (2019). Trends in the incidence of testing for vitamin D deficiency in primary care in the UK: a retrospective analysis of The Health Improvement Network (THIN), 2005–2015. BMJ Open.

[2] Cashman KD (2020). Vitamin D deficiency: defining, prevalence, causes, and strategies of addressing. Calcif Tissue Int.

[3] Sempos CT, Vesper HW, Phinney KW, Thienpont LM, Coates PM (2012). Vitamin D Standardization Program (VDSP). Vitamin D status as an international issue: national surveys and the problem of standardization. Scand J Clin Lab Invest Suppl.

[4] Ross AC, Taylor CL, Yaktine AL, Del Valle HB, Institute of Medicine (US) (2011). Committee to review dietary reference intakes for vitamin D and calcium. Dietary reference intakes for calcium and vitamin D.

[5] EFSA Panel on Dietetic Products, Nutrition and Allergies (NDA) (2016). Dietary reference values for vitamin D. EFSA J.

[6] GOV.UK Scientific Advisory Committee on Nutrition (SACN). SACN vitamin D and health report [Internet]. ..

[7] Holick MF, Binkley NC, Bischoff-Ferrari HA, Gordon CM, Hanley DA, Heaney RP (2011). Evaluation, treatment, and prevention of vitamin D deficiency: an Endocrine Society clinical practice guideline. J Clin Endocrinol Metab.

[8] Vieth R (2006). What is the optimal vitamin D status for health?. Prog Biophys Mol Biol.

[9] Dawson-Hughes B, Mithal A, Bonjour J-P, Boonen S, Burckhardt P, Fuleihan GE-H (2010). IOF position statement: vitamin D recommendations for older adults. Osteoporos Int.

[10] American Geriatrics Society Workgroup on Vitamin D Supplementation for Older Adults (2014). Recommendations abstracted from the American Geriatrics Society Consensus Statement on vitamin D for prevention of falls and their consequences. J Am Geriatr Soc.

[11] Cosman F, de Beur SJ, LeBoff MS, Lewiecki EM, Tanner B, Randall S (2015). Erratum to: clinician’s guide to prevention and treatment of osteoporosis. Osteoporos Int.

[12] Cashman KD, Kiely M (2011). Towards prevention of vitamin D deficiency and beyond: knowledge gaps and research needs in vitamin D nutrition and public health. Br J Nutr.

[13] Binkley N, Krueger D, Cowgill CS, Plum L, Lake E, Hansen KE (2004). Assay variation confounds the diagnosis of hypovitaminosis D: a call for standardization. J Clin Endocrinol Metab.

[14] Carter GD, Carter R, Jones J, Berry J (2004). How accurate are assays for 25-hydroxyvitamin D? Data from the international vitamin D external quality assessment scheme. Clin Chem.

[15] Carter GD (2012). 25-hydroxyvitamin D: a difficult analyte. Clin Chem.

[16] Lai JKC, Lucas RM, Banks E, Ponsonby A-L, Ausimmune Investigator Group (2012). Variability in vitamin D assays impairs clinical assessment of vitamin D status. Intern Med J.

[17] Schleicher RL, Sternberg MR, Looker AC, Yetley EA, Lacher DA, Sempos CT (2016). National estimates of serum total 25-hydroxyvitamin D and metabolite concentrations measured by liquid chromatography-tandem mass spectrometry in the US population during 2007–2010. J Nutr.

[18] Sarafin K, Durazo-Arvizu R, Tian L, Phinney KW, Tai S, Camara JE (2015). Standardizing 25-hydroxyvitamin D values from the Canadian Health Measures Survey. Am J Clin Nutr.

[19] Cashman KD, Dowling KG, Škrabáková Z, Gonzalez-Gross M, Valtueña J, De Henauw S (2016). Vitamin D deficiency in Europe: pandemic?. Am J Clin Nutr.

[20] Saliba W, Barnett O, Rennert HS, Lavi I, Rennert G (2011). The relationship between serum 25(OH)D and parathyroid hormone levels. Am J Med.

[21] Sai AJ, Walters RW, Fang X, Gallagher JC (2011). Relationship between vitamin D, parathyroid hormone, and bone health. J Clin Endocrinol Metab.

[22] Souberbielle JC, Cormier C, Kindermans C, Gao P, Cantor T, Forette F (2001). Vitamin D status and redefining serum parathyroid hormone reference range in the elderly. J Clin Endocrinol Metab.

[23] Sahota O, Mundey MK, San P, Godber IM, Lawson N, Hosking DJ (2004). The relationship between vitamin D and parathyroid hormone: calcium homeostasis, bone turnover, and bone mineral density in postmenopausal women with established osteoporosis. Bone.

[24] Gutiérrez OM, Farwell WR, Kermah D, Taylor EN (2011). Racial differences in the relationship between vitamin D, bone mineral density, and parathyroid hormone in the National Health and Nutrition Examination Survey. Osteoporos Int.

[25] Torregrosa J-V, Bover J, Cannata Andía J, Lorenzo V, de Francisco ALM, Martínez I (2011). Recomendaciones de la Sociedad Española de Nefrología para el manejo de las alteraciones del metabolismo óseo-mineral en los pacientes con enfermedad renal crónica (S.E.N.-M.M.). Nefrología.

[26] Bolland MJ, Chiu WW, Davidson JS, Grey A, Bacon C, Gamble GD (2008). The effects of seasonal variation of 25-hydroxyvitamin D on diagnosis of vitamin D insufficiency. N Z Med J.

[27] Aguado P, del Campo MT, Garcés MV, González-Casaús ML, Bernad M, Gijón-Baños J (2000). Low vitamin D levels in outpatient postmenopausal women from a rheumatology clinic in Madrid, Spain: their relationship with bone mineral density. Osteoporos Int.

[28] Quesada-Gómez JM, Diaz-Curiel M, Sosa-Henriquez M, Malouf-Sierra J, Nogues-Solan X, Gomez-Alonso C (2013). Low calcium intake and inadequate vitamin D status in postmenopausal osteoporotic women. J Steroid Biochem Mol Biol.

[29] Mata-Granados JM, Luque de Castro MD, Quesada Gomez JM (2008). Inappropriate serum levels of retinol, alpha-tocopherol, 25 hydroxyvitamin D3 and 24,25 dihydroxyvitamin D3 levels in healthy Spanish adults: simultaneous assessment by HPLC. Clin Biochem.

[30] Lips P, Duong T, Oleksik A, Black D, Cummings S, Cox D (2001). A global study of vitamin D status and parathyroid function in postmenopausal women with osteoporosis: baseline data from the multiple outcomes of raloxifene evaluation clinical trial. J Clin Endocrinol Metab.

[31] Larrosa M, Gratacòs J, Vaqueiro M, Prat M, Campos F, Roqué M (2001). Prevalence of hypovitaminosis D in elderly institutionalized residents: influence of a substitutive treatment. Med Clin (Barc).

[32] Vaqueiro M, Baré ML, Anton E, Andreu E, Gimeno C, D’AVIS Group (2006). Evaluation assessment of the cut-off point of vitamin D in the population older than 64 years old. Med Clin (Barc).

[33] AlQuaiz AM, Kazi A, Fouda M, Alyousefi N (2018). Age and gender differences in the prevalence and correlates of vitamin D deficiency. Arch Osteoporos.

[34] Vallejo MS, Blümel JE, Arteaga E, Aedo S, Tapia V, Araos A (2020). Gender differences in the prevalence of vitamin D deficiency in a southern Latin American country: a pilot study. Climacteric.

[35] Bringhurst FR, Demay MB, Krane SM, Kronenberg HM, Kasper D, Fauci A, Hauser S, Longo D, Jameson JL, Loscalzo J (2014). Bone and mineral metabolism in health and disease. Harrison’s principles of internal medicine.

[36] (2021). Vitamin D deficiency and related disorders: practice essentials, background. Pathophysiology.

[37] Pereda CA, Nishishinya MB, Roldan EJA (2019). 25-Hydroxyvitamin D serum levels in rheumatic female patients in southeast Spain: the paradigm of daily optimal sunshine levels and inadequate vitamin D status. Endocrinol Diabetes Nutr.

[38] Gozdzik A, Barta JL, Weir A, Cole DEC, Vieth R, Whiting SJ (2010). Serum 25-hydroxyvitamin D concentrations fluctuate seasonally in young adults of diverse ancestry living in Toronto. J Nutr.

[39] Itoh H, Mori I, Matsumoto Y, Maki S, Ogawa Y (2011). Vitamin D deficiency and seasonal and inter-day variation in circulating 25-hydroxyvitamin D and parathyroid hormone levels in indoor daytime workers: a longitudinal study. Ind Health.

[40] Holick MF (2004). Sunlight and vitamin D for bone health and prevention of autoimmune diseases, cancers, and cardiovascular disease. Am J Clin Nutr.

[41] Li M, Lv F, Zhang Z, Deng W, Li Y, Deng Z (2016). Establishment of a normal reference value of parathyroid hormone in a large healthy Chinese population and evaluation of its relation to bone turnover and bone mineral density. Osteoporos Int.

[42] Shen M, Li Z, Lv D, Yang G, Wu R, Pan J (2020). Seasonal variation and correlation analysis of vitamin D and parathyroid hormone in Hangzhou, Southeast China. J Cell Mol Med.

